# Effect of previous heterologous flavivirus vaccinations on human antibody responses in tick‐borne encephalitis and dengue virus infections

**DOI:** 10.1002/jmv.29245

**Published:** 2023-11-27

**Authors:** Lena Roßbacher, Stefan Malafa, Kristina Huber, Melissa Thaler, Stephan W. Aberle, Judith H. Aberle, Franz X. Heinz, Karin Stiasny

**Affiliations:** ^1^ Center for Virology Medical University of Vienna Vienna Austria; ^2^ Division of Infectious Diseases and Tropical Medicine University Hospital, LMU Munich Munich Germany; ^3^ Present address: Department of Medical Microbiology Leiden University Medical Center Leiden The Netherlands

**Keywords:** dengue virus, flavivirus antibody cross‐reactivity, flavivirus maturation, flavivirus neutralization, structural dynamics, tick‐borne encephalitis virus

## Abstract

Arthropod‐borne flaviviruses include a number of medically relevant human pathogens such as the mosquito‐borne dengue (DEN), Zika, and yellow fever (YF) viruses as well as tick‐borne encephalitis virus (TBEV). All flaviviruses are antigenically related and anamnestic responses due to prior immunity can modulate antibody specificities in subsequent infections or vaccinations. In our study, we analyzed the induction of broadly flavivirus cross‐reactive antibodies in tick‐borne encephalitis (TBE) and DEN patients without or with prior flavivirus exposure through TBE and/or YF vaccination, and determined the contribution of these antibodies to TBE and dengue virus (DENV) neutralization. In addition, we investigated the formation of cross‐reactive antibodies in TBE‐vaccination breakthroughs (VBTs). A TBEV infection without prior YF or TBE vaccination induced predominantly type‐specific antibodies. In contrast, high levels of broadly cross‐reactive antibodies were found in samples from TBE patients prevaccinated against YF as well as in DEN patients prevaccinated against TBE and/or YF. While these cross‐reactive antibodies did not neutralize TBEV, they were effective in neutralizing DENV. This discrepancy points to structural differences between the two viruses and indicates that broadly cross‐reactive epitopes are less accessible in TBEV than in DENV. In TBE VBT infections, type‐specific antibodies dominated the antibody response, thus revealing no difference from that of unvaccinated TBE patients. Our results emphasize significant differences in the structural properties of different flaviviruses that have an impact on the induction of broadly cross‐reactive antibodies and their functional activities in virus neutralization.

## 1 INTRODUCTION

Flaviviruses comprise important arthropod‐borne human pathogens including the mosquito‐borne dengue (DEN), Zika, and yellow fever (YF) viruses as well as tick‐borne encephalitis virus (TBEV).^1^ The potential of sequential human exposures to antigens of various flaviviruses has increased due to their widespread distribution, co‐circulation in geographical regions, people's mobility, and vaccine use. Since all flaviviruses are antigenically related, previous contacts with conserved sequence elements in heterologous flavivirus antigens may lead to anamnestic immune responses affecting extent and antibody patterns during subsequent flavivirus infections and/or vaccinations.^2^, ^3^, ^4^

**Figure 1:**
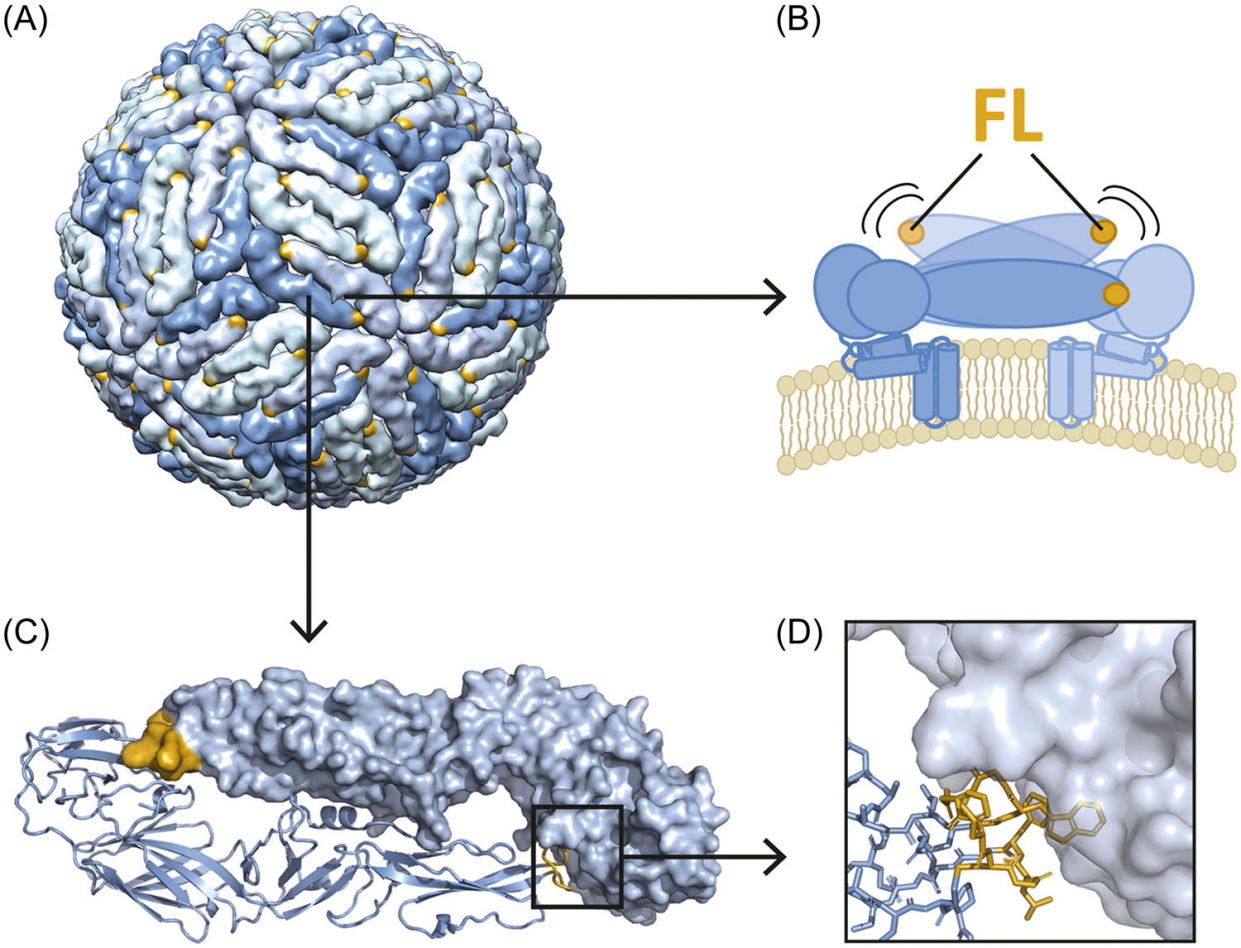
Structural organization of tick‐borne encephalitis virus (TBEV). (A) Mature TBEV particle (PDB 5O6A^18^) with E dimers organized in a herringbone‐like shell. (B) Schematic representation of the dynamics (“breathing”) of an E dimer (side view) leading to the transient exposure of the fusion loop (FL, dark yellow). (C) Representation of the E dimer (top view) of TBEV (PDB 1SVB^19^) and (D) zoom of the FL buried at the dimer interface. The panels showing the structures were made with UCSF Chimera (www.rbvi.ucsf.edu/chimera) and PyMol (www.pymol.org).

Structurally, all flaviviruses are very similar with respect to the molecular organization and maturation pathways of viral particles.^5^ The major envelope protein E covers the surface of mature virions in a herringbone‐like pattern (Figure 1A)^6^ and, because of its viral entry functions is the target of neutralizing and protective antibodies. This protein exhibits only ∼40% amino acid identity between distantly related flaviviruses,^2^ like TBEV and dengue DEN virus (DENV) or YF virus (YFV), and most of its surface‐exposed amino acids are variable. However, E can undergo dynamic motions (“virus breathing,” Figure 1B) that lead to the transient exposure of conserved internal structural elements,^7^ like the otherwise buried fusion loop (FL) (Figure 1B–D), which gives rise to broadly flavivirus cross‐reactive antibodies.^8^, ^9^, ^10^ The extent of breathing and concomitant FL exposure varies among flaviviruses,^8^, ^11^ with the substantially more stable TBEV being less prone to FL exposure than the more dynamic DENV.^12^ Differences in the display of antigenic sites, including the FL, during flavivirus infections, may also result from inefficient maturation of immature precursor particles and the formation of only partially mature virions,^13^, ^14^ a phenomenon that appears to be especially prominent with DENV.^15^, ^16^, ^17^

In sequential exposures to distantly related flaviviruses, the FL and other conserved sequence elements can elicit anamnestic responses and boosting of cross‐reactive antibodies.^3^, ^4^ FL‐specific antibodies have been described to be not or only weakly neutralizing^8^ but are implicated in contributing to severe forms of DEN by increasing virus infection of Fc‐gamma‐receptor‐positive cells via antibody‐dependent enhancement (ADE).^20^

Formalin‐inactivated TBE vaccines are widely used in European countries, where TBEV is endemic.^21^ These vaccines exhibit a high field effectiveness of >90% and led to a dramatic decline in cases in countries with high vaccination coverage such as Austria.^22^ Vaccination‐breakthrough (VBT) infections have been reported rarely and were shown to be associated with strong anamnestic immune responses in most instances.^23^, ^24^, ^25^ It is not yet clear, whether the epitope specificity of anamnestic responses in VBTs differs from that after natural infections without previous vaccination and might support speculations about immune‐enhancement phenomena in VBT situations.^26^

European travelers to YF‐endemic regions are often vaccinated with the live‐attenuated YF vaccine,^27^ and many return from tropical and subtropical regions with DENV infections.^28^

In this study, we investigated the effects of anamnestic responses on antibody formation in TBE and DEN patient cohorts with or without a history of YF and/or TBE vaccination, including broadly flavivirus cross‐reactive antibodies, and assessed their contribution to virus neutralization. We found that TBE patients without a prior TBE or YF vaccination developed predominantly type‐specific antibodies, whereas the response in YF‐prevaccinated TBE as well as YF‐ and/or TBE‐prevaccinated DEN patients was skewed toward cross‐reactive antibodies. TBEV neutralization was not affected by broadly cross‐reactive antibodies, but the same antibodies neutralized DENV, suggesting differences in the accessibility of broadly cross‐reactive epitopes in different flaviviruses, which can be related to structural differences between TBE and DEN viruses. In TBE VBTs, we did not observe a shift toward cross‐reactive antibodies. Rather, the antibody response was dominated by type‐specific antibodies, like in infections of unvaccinated TBE patients.

## 2 MATERIALS AND METHODS

### 2.1 Human serum samples

Human sera were originally sent for diagnostic purposes to the Center for Virology, Medical University of Vienna, Austria. In this retrospective study, only leftover anonymized samples were analyzed, with the approval of the ethics committee of the Medical University of Vienna (EK 1184/2020).

Serum samples of confirmed unvaccinated TBE cases were available from 30 patients, with a previous YF vaccination from 11, and with previous TBE vaccinations (VBT) from 11 patients. All patients developed TBEV‐specific immunoglobulin M (IgM) and IgG antibodies, which were quantified with in‐house enzyme‐linked immunosorbent assays (ELISAs) using purified whole virus and/or soluble E (sE) as antigens, as described previously.^29^, ^30^ Serum samples of polymerase chain reaction (PCR)‐confirmed DEN cases with prior TBE and/or YF vaccination were available from 27 patients (5 DENV‐serotype 1, 22 DENV‐serotype 2). These patients were travelers returning from DEN‐endemic regions.

### 2.2 DEN PCR and serotyping

DENV serotypes were determined as described previously.^30^ Briefly, viral nucleic acids were extracted automatically from serum samples (NucliSens easyMAG platform; Biomerieux), according to the manufacturer's instructions. DEN RNA TaqMan‐based real‐time PCR was performed with primers targeting the 3′ noncoding region.^31^ Serotyping was carried out with the ArboTyping software.^32^ Validation was done with proficiency panels from the European Centre for Disease Prevention and Control, the Emerging and Vector Borne Laboratory Network, and the Royal College of Pathologists of Australasia Quality Assurance Program and World Health Organization.

### 2.3 Confirmation of prior vaccinations by TBE and YF neutralization tests (NTs)

TBE and YF NTs were performed as described previously.^12^, ^30^ Briefly, serially diluted serum samples were mixed with TBEV strain Neudoerfl^33^, ^34^ or YFV strain 17D.^35^ BHK‐21 cells (ATCC) were added and incubated for 3 (TBEV) or 4 days (YFV). YF NT titers were expressed as the reciprocal of the serum dilution that prevented the development of a virus‐induced cytopathic effect (CPE). NT titers ≥ 20 were considered positive. Since TBEV strain Neudoerfl does not produce a CPE of BHK cells, inhibition of virus replication was determined by measuring the presence of the virus in the cell culture supernatants by ELISA.^36^, ^37^ The NT titer was defined as the reciprocal of the serum dilution that gave a 90% reduction in the absorbance readout in the assay compared with the control without antibodies. NT titers ≥ 10 were considered positive.

### 2.4 Production of recombinant flavivirus sE proteins

Recombinant sE proteins, lacking the membrane‐anchoring regions, with a C‐terminal double Strep‐Tag were produced with the Drosophila‐Expression System (Invitrogen), as described previously.^12^, ^38^ Briefly, protein expression in stably transfected Schneider S2 cells was induced by the addition of CuSO_4_, and recombinant sEs were purified by Strep‐Tactin‐affinity chromatography (IBA GmbH), according to the manufacturers' instructions. The sequences of the different sEs (GeneArt; ThermoFisher Scientific) were based on the following Genbank accession codes: U27495 (TBEV), AF226687.2 (DENV‐serotype 1), NC_001474.2 (DENV‐serotype 2), and AF144692.1 (Rio Bravo virus, RBV).

### 2.5 Flavivirus IgG ELISA

IgG ELISAs were performed as described previously.^12^, ^30^ Serially diluted serum samples were added to flavivirus sE proteins (25 ng/well) captured via their Strep‐Tag to Strep‐Tactin‐coated microplates (IBA GmbH). Bound IgG antibodies were detected with a goat‐anti‐human‐IgG‐horseradish‐peroxidase conjugate (Thermo Fisher Scientific). Antibody titers were determined by curve fitting with a four‐parameter logistic regression (GraphPad Prism 9) using a cutoff of the mean absorbance at the starting dilution plus three standard deviations of 32 flavivirus‐negative diagnostic serum samples from previous studies^12^ (Figure S1A). Each serum was tested at least twice and geometric mean titers (GMTs) were calculated.

This assay allows the presentation of the sE proteins in identical form and preserves their native structure.^12^ As shown in a previous study, broadly cross‐reactive antibodies yielded the same reactivities with sE proteins, from different flaviviruses in this format.^12^ We retested a broadly cross‐reactive serum, used as a positive control in the previous study (from a Zika patient with prior YF and TBE vaccination), with the sE antigens of DEN1, DEN2, TBE, and RBVs and included it on each plate and in each ELISA to control for assay variations. We obtained identical titration curves of this broadly cross‐reactive serum with all sE proteins tested (Figure S1B).

### 2.6 Flavivirus NTs

NTs were performed as described previously^12^ using TBEV (strain Neudoerfl), DENV‐serotype 1 (strain Hawaii, kindly provided by Herbert Schmitz; Bernhard‐Nocht‐Institute), or DENV‐serotype 2 (strain NGC, European Virus Archive). Serial serum dilutions were preincubated with the respective virus for 1 h at 37°C. BHK‐21 (TBEV) or Vero cells (DENV) were added and after 3 or 4 days at 37°C, respectively, cells were fixed with 4% paraformaldehyde. Mouse‐monoclonal antibody IC3, recognizing TBEV,^39^ or 2H2 (ATCC HB114), recognizing DENV,^40^, ^41^ was used for detection together with a rabbit‐anti‐mouse‐Alexa‐Fluor‐488‐labeled secondary antibody (Invitrogen). Titers were determined by curve fitting with a four‐parameter logistic regression (GraphPad Prism 9) using a 50% fluorescence reduction in the absence of antibody as cutoff (NT50). Each serum was tested at least twice and GMTs were calculated.

### 2.7 Depletion of antibodies

Depletion of flavivirus broadly cross‐reactive antibodies was done as previously described.^12^ 200 μg Rio Bravo (RB) sE were bound to Strep‐Tactin‐XT spin columns (IBA GmbH), following the manufacturer's protocol. Prediluted samples (1:5) were loaded onto the columns, incubated for 10 min, and centrifuged according to the manufacturer's instructions. At least eight rounds of depletion were necessary to completely remove RB‐sE‐binding antibodies. Depletion was confirmed by IgG ELISA with RB sE as antigen. Mock depletion was performed with Strep‐Tactin‐XT spin columns in the absence of sE.

### 2.8 Statistics

Statistical analyses were performed with GraphPad Prism 9 (GraphPad Software Inc.). Significance testing was done with two‐tailed *t* tests or one‐way analysis of variance with Bonferroni's multiple comparison test for log‐transformed titers, and Mann–Whitney tests or Kruskal–Wallis tests followed by Dunn's multiple comparison tests for ratios. Correlation coefficients were determined with the Pierson correlation test. *p* ≤ 0.05 were considered significant.

## 3 RESULTS

### 3.1 Study cohorts

Serum samples were obtained from 41 TBE patients after hospitalization with neurological symptoms (Table 1), that is, in the second phase of the disease (median of 14 days after disease onset, Table S1). Thirty patients had no prior TBE or YF vaccination (unvaccinated cohort) and 11 patients had a previous YF vaccination (Table 1). Serum samples from 27 DEN patients (5 DENV‐serotype 1, 22 DENV‐serotype 2) were obtained at a median of 8 days after diagnosis by PCR and a median of 11 days after disease onset (Table S2). All DEN patients had a history of flavivirus vaccination, against either TBE, YF, or both (Table 2). Further characteristics of TBE and DEN patients are summarized in Tables 1 and 2, respectively.

**Table 1:** Characteristics of TBE patients with and without prior heterologous flavivirus vaccination.

TBE patients	No. of cases	Age (years)		Sex (f/m)
		Median	Range	
Unvaccinated	30	55	3–82	11/19
Prior YF vaccination^a^	11	53	26–75	3/8

Abbreviations: f, female; m, male; TBE, tick‐borne encephalitis; YF, yellow fever.

^a^ Time point of YF vaccination not reported to the diagnostic laboratory, confirmed by YF neutralization test.

**Table 2:** Characteristics of DEN patients with prior TBE and/or YF vaccination.

DEN patients	No. of cases	Age (years)		Sex (f/m)
		Median	Range	
Prior TBE vaccination^a^	14	29	14–62	9/5
Prior YF vaccination^a^	2	48	37–58	1/1
Prior TBE + YF vaccination^a^	11	48	23–58	8/3

Abbreviations: DEN, dengue; f, female; m, male; TBE, tick‐borne encephalitis; YF, yellow fever.

^a^ Time point of TBE and YF vaccination not reported to the diagnostic laboratory, confirmed by TBE and YF neutralization tests.

### 3.2 Comparison of broadly cross‐reactive antibodies after TBEV and DENV infections in individuals with prior heterologous flavivirus immunity

**Figure 2:**
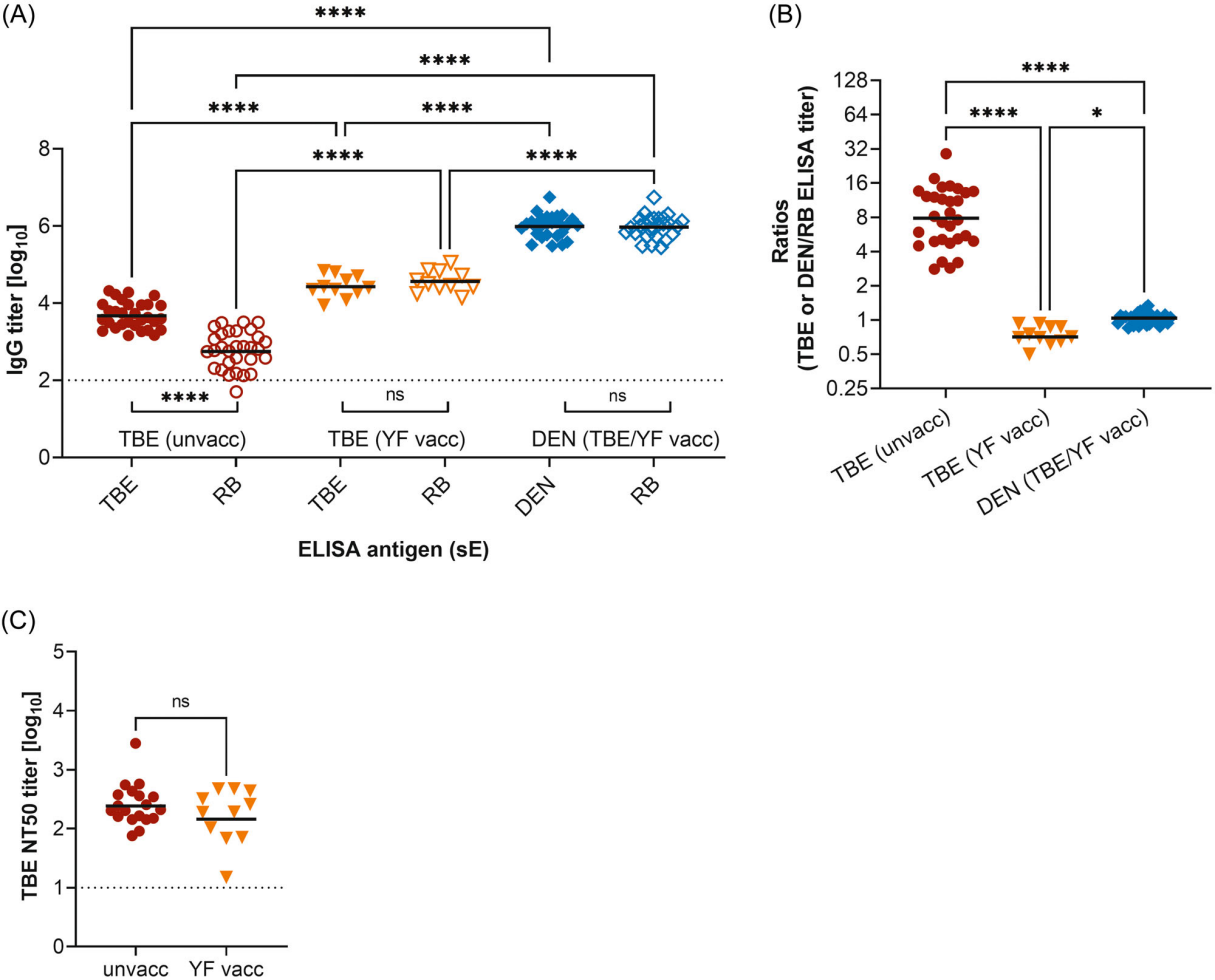
Flavivirus‐specific IgG antibody titers of tick‐borne encephalitis (TBE) and dengue (DEN) postinfection sera. (A) IgG titers, determined with the respective homologous sE (TBE or DEN), are shown as filled symbols, broadly cross‐reactive IgG titers, determined with Rio Bravo (RB) sE, as empty symbols. (B) Ratios of homologous to broadly cross‐reactive antibody titers. Red circles: TBE patients without prior TBE or YF vaccination (unvacc, *n* = 30); orange triangles: TBE patients with a prior YF vaccination (YF vacc, *n* = 11); blue diamonds: DEN patients with prior TBE and/or YF vaccination (TBE/YF vacc, *n* = 27). (C) TBE neutralizing antibody titers (NT). Red circles: TBE patients without a prior TBE or YF vaccination (unvacc, *n* = 19); orange triangles: TBE patients with a prior YF vaccination (YF vacc, *n* = 11). Bars show the GMT (A, C) or median titer ratios (B). Among the three groups, significant differences between ELISA titers were determined by one‐way ANOVA followed by Bonferroni's multiple comparison test (A) and between ELISA‐titer ratios with the Kruskal–Wallis test followed by Dunn's multiple comparison test (B). (C) *t* tests were used for the comparison of NT titers. Significant differences within the groups (A) were determined by *t* tests. Significances are indicated by asterisks (****p < 0.0001, *p = 0.01–0.05). Dotted line: a cutoff of the assays. ANOVA, analysis of variance; DEN, dengue; ELISA, enzyme‐linked immunosorbent assays; GMT, geometric mean titer; IgG, immunoglobulin G; ns, not significant; sE, soluble E; TBE, tick‐borne encephalitis; unvacc, unvaccinated; vacc, vaccinated; YF, yellow fever.

Flavivirus‐specific IgG antibodies were quantified by ELISAs in sera of unvaccinated and YF‐prevaccinated TBE patients as well as of TBE and/or YF‐prevaccinated DEN patients (Tables 1 and 2). We used the sE protein of the infecting virus (homologous sE of TBEV or the respective DENV serotype) for determining overall E‐specific antibodies and the sE of RBV (a distantly related flavivirus) for selectively determining broadly cross‐reactive antibodies (Figure 2A), as described in Section 2.

In unvaccinated TBE patients, only low levels of cross‐reactive antibodies were detected and antibody titers against the homologous sE were significantly higher (Figure 2A), resulting in a high ratio (∼eightfold) of homologous to cross‐reactive antibody titers (Figure 2B). In contrast, YF‐prevaccinated TBE patients displayed a dramatically different pattern, with similar high levels of homologous and cross‐reactive antibody titers (Figure 2A); the corresponding ratio therefore was close to 1 (Figure 2B). Since ELISAs with the homologous sE protein measures both type‐specific and broadly cross‐reactive antibodies,^12^, ^42^ we also determined TBE NT titers, which are type‐specific and thus can allow conclusions as to whether pre‐existing flavivirus immunity would also result in a booster of neutralizing antibodies. There was no significant difference between the two groups (Figure 2C), indicating that the YF preimmunity mainly caused a shift in the antibody repertoire toward nonneutralizing and broadly cross‐reactive antibodies.

DEN patients with previous TBE and/or YF vaccination exhibited even higher antibody titers than YF‐prevaccinated TBE patients, both in the ELISA with the homologous DEN and the heterologous RB sE (Figure 2A). There was no significant difference between the two categories, resulting in ELISA‐titer ratios of approximately one (Figure 2B). We stratified DEN patients with respect to their prior exposure history through TBE and/or YF vaccination or the infecting DENV serotype and found no statistical differences between the different groups, as shown in Figure S2.

### 3.3 Neutralizing activity of cross‐reactive antibodies

To determine the contribution of cross‐reactive antibodies to virus neutralization in serum samples of the different cohorts, we carried out depletion experiments with pools of equal aliquots of sera, for which at least 500 µL were available (Table S3). The mean antibody titers of these sera matched those obtained with the pools in the respective IgG ELISAs (Figure S3). The pools were depleted with RB sE (Section 2), and TBEV and DENV neutralization was determined before and after depletion.

**Figure 3:**
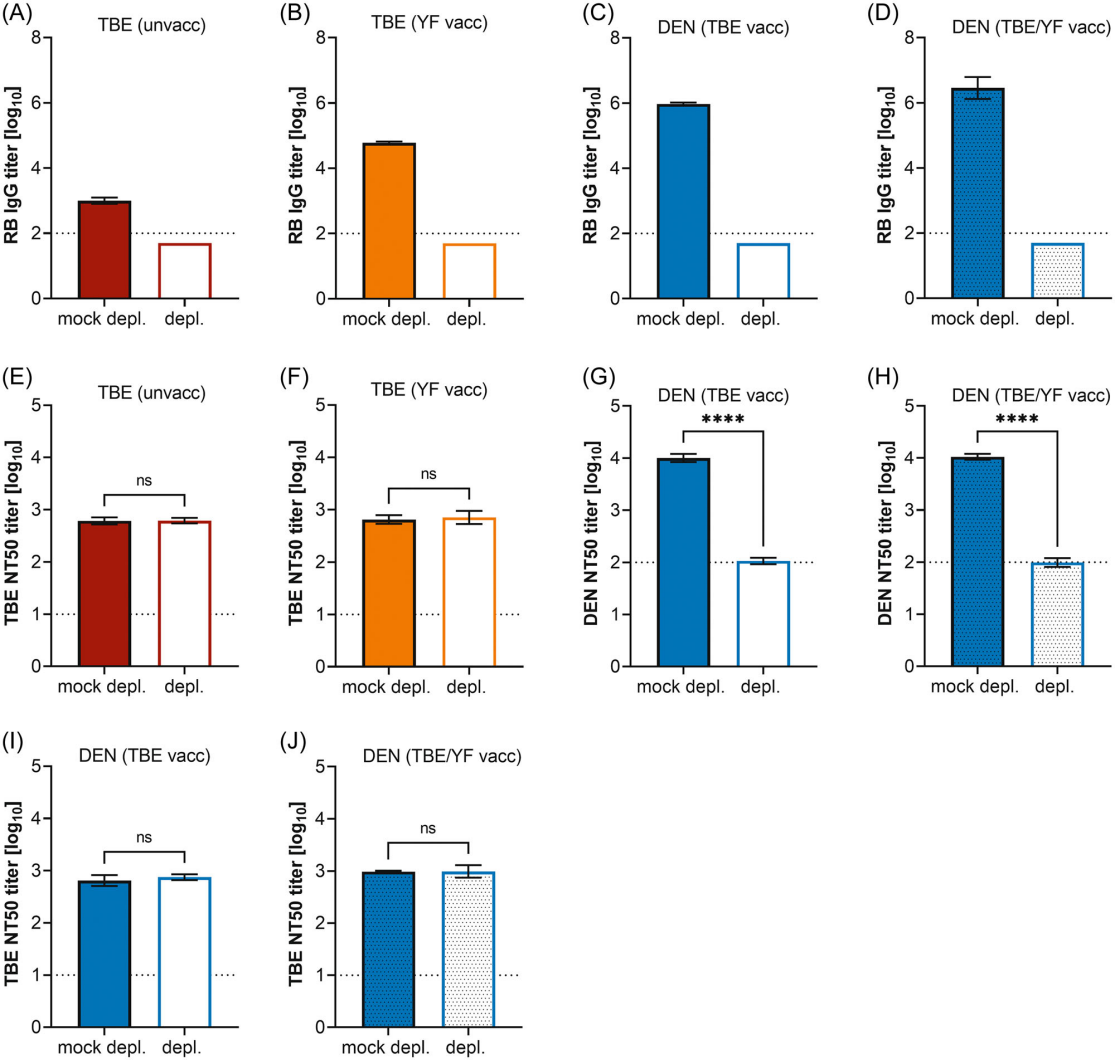
Contribution of broadly cross‐reactive antibodies to virus neutralization in serum pools from TBE and DEN patients. TBE patients without a prior TBE or YF vaccination (TBE [unvacc]), TBE patients with a prior YF vaccination (TBE [YF vacc]), DEN patients with a prior TBE vaccination (DEN [TBE vacc]), and DEN patients with a prior TBE and YF vaccination (DEN [TBE/YF vacc]). (A–D) RB IgG antibody titers of serum pools after depletion of broadly cross‐reactive antibodies. (E–J) Neutralizing antibody titers of serum pools after depletion of broadly cross‐reactive antibodies with RB sE. (E) TBEV‐neutralizing antibody titers of the pool from TBE patients without a prior TBE or YF vaccination. (F) TBEV‐neutralizing antibody titers of the pool from TBE patients with a prior YF vaccination. (G) DENV‐neutralizing antibody titers of the pool from DEN patients with a prior TBE vaccination. (H) DENV‐neutralizing antibody titers of the pool from DEN patients with a prior TBE and YF vaccination. (I) TBEV‐neutralizing antibody titers of the pool from DEN patients with a prior TBE vaccination. (J) TBEV‐neutralizing antibody titers of the pool from DEN patients with a prior TBE and YF vaccination. The pools were tested in three independent experiments. Significances were determined with *t* tests and are indicated with asterisks (****p < 0.0001). Error bars show the standard error of the mean. Dotted line: cutoff of the assay. DEN, dengue; depl., depleted serum pools; IgG, immunoglobulin G; mock depl., mock‐depleted serum pools; ns, not significant; RB, Rio Bravo; sE, soluble E; TBE, tick‐borne encephalitis; TBEV, tick‐borne encephalitis virus; unvacc, unvaccinated; vacc, vaccinated; YF, yellow fever.

As shown in Figure 3A–D, the depletion procedure was quantitative and resulted in the loss of >99% RB sE reactivity of each pool. In the two pools of TBE patients, the depletion of cross‐reactive antibodies had no effect on TBEV neutralization (Figure 3E,F). In contrast, the pools from TBE‐ and/or YF‐prevaccinated DEN patients displayed a dramatic decline of DENV‐neutralizing activity after depletion (Figure 3G,H), indicating that neutralization was almost completely exerted by cross‐reactive antibodies. Consistent with these findings, TBEV‐type‐specific neutralization activity (resulting from previous TBE vaccination) was completely unaffected by depleting cross‐reactive antibodies from the DEN serum pools (Figure 3I,J).

**Figure 4:**
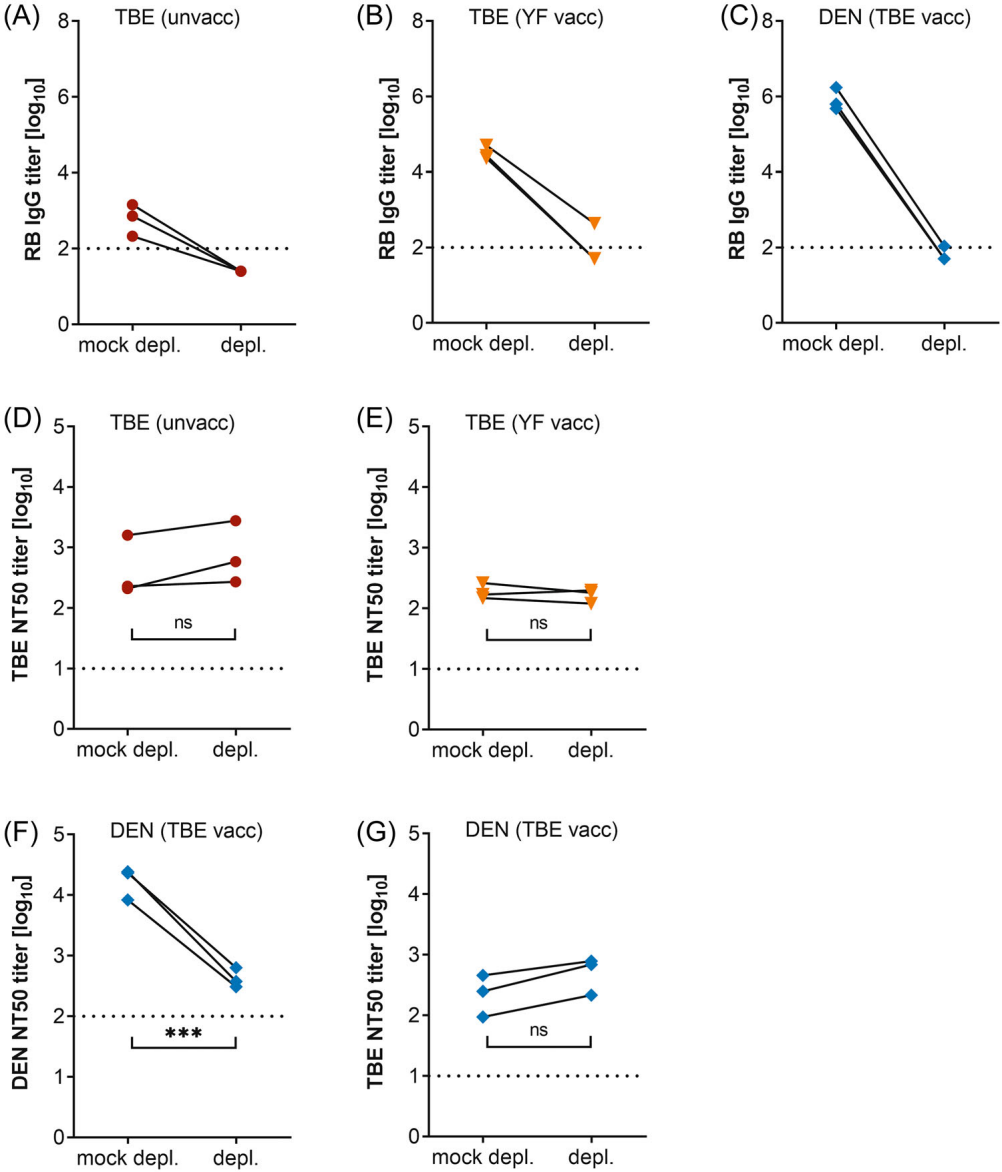
Contribution of broadly cross‐reactive antibodies to virus neutralization in individual sera from TBE and DEN patients. Three individual sera per group were depleted with RB sE. TBE patients without a prior TBE or YF vaccination (unvaccinated [unvacc]), TBE patients with a prior YF vaccination (TBE [YF vacc]), and DEN patients with a prior TBE vaccination (DEN [TBE vacc]). (A–C) RB IgG antibody titers of sera after depletion of broadly cross‐reactive antibodies with RB sE. (D‐G) Neutralizing antibody titers of sera after depletion of broadly cross‐reactive antibodies with RB sE. (D) TBEV‐neutralizing antibody titers of sera from unvaccinated TBE patients. (E) TBEV‐neutralizing antibody titers of sera from TBE patients with a prior YF vaccination. (F) DENV‐neutralizing antibody titers of sera from DEN patients with a prior TBE vaccination. (G) TBEV‐neutralizing antibody titers of sera from DEN patients with a prior TBE vaccination. Significances were determined with *t* tests and are indicated with asterisks (***p = 0.0001–0.001). Dotted line: cutoff of the assay. DEN, dengue; depl., depleted sera; IgG, immunoglobulin G; mock depl., mock‐depleted sera; ns, not significant; NT, neutralization test; RB, Rio Bravo; sE, soluble E; TBE, tick‐borne encephalitis; TBEV, tick‐borne encephalitis virus; vacc, vaccinated; YF, yellow fever.

To identify possible individual variation relative to the findings obtained with the serum pools, we conducted depletion experiments with three representative sera from each of the cohorts (Figure 4). The selection was based on the availability of (i) sufficient sample volumes and (ii) ELISA‐titer ratios (virus‐type specific to cross‐reactive antibodies, Figure 2B) within the 25% and 75% percentiles of all sera (Figure 2B). As for the pools, RB‐sE‐depletion was quantitative (Figure 4A–C), and the NT results with single samples reflected those obtained with the pools (Figure 4D–G); that is, depleting broadly cross‐reactive antibodies had no effect on TBEV neutralization (Figure 4D,E,G), but substantially reduced DENV neutralization (Figure 4F).

### 3.4 Broadly cross‐reactive antibodies in TBE vaccination‐breakthrough infections

**Figure 5:**
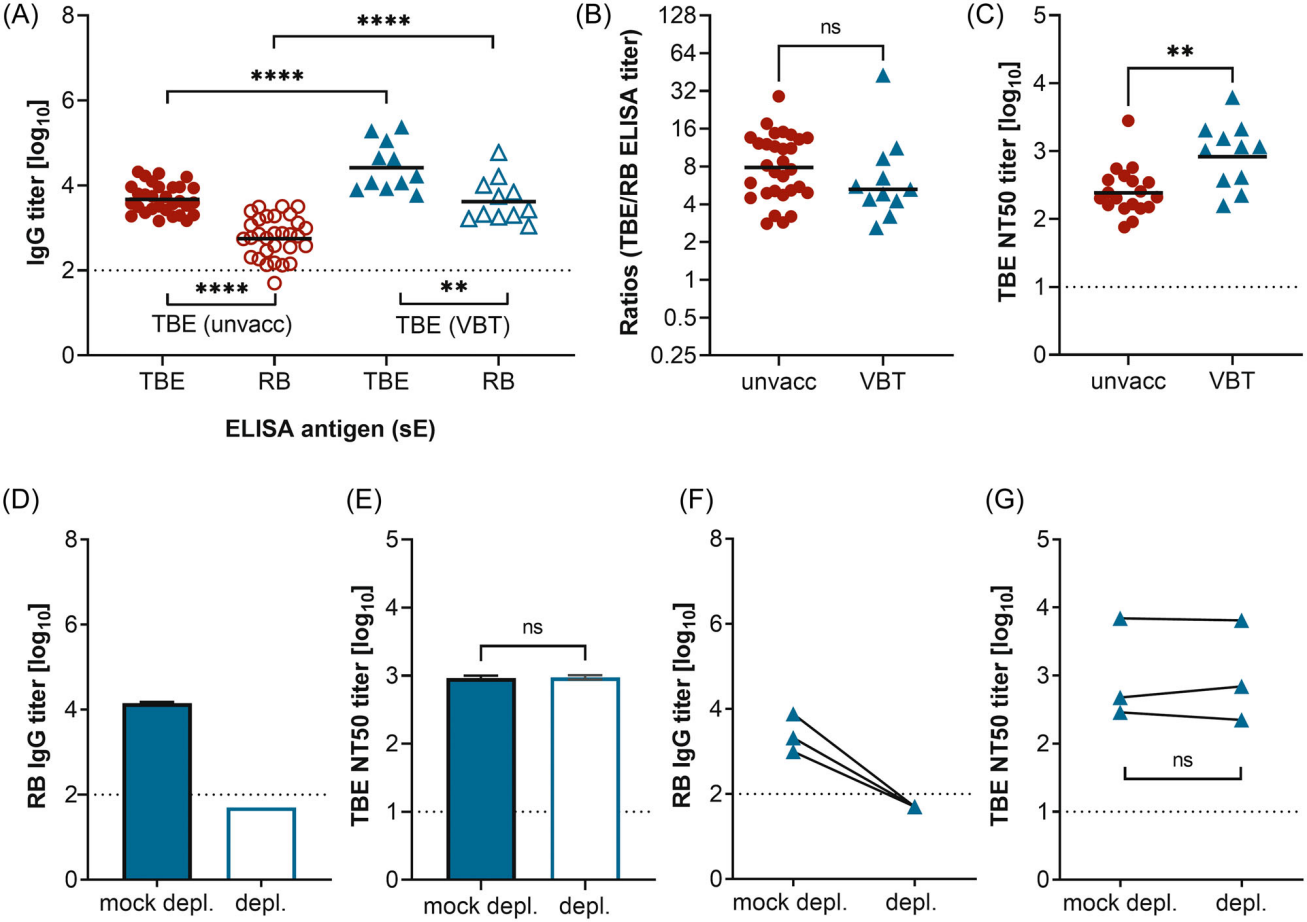
Flavivirus‐specific IgG antibody titers of TBE vaccination‐breakthrough (VBT) sera. (A) Type‐specific IgG titers, determined with TBE sE, are shown as filled symbols, and broadly cross‐reactive IgG titers, determined with Rio Bravo (RB) sE, as empty symbols. (B) Ratios of type‐specific to broadly cross‐reactive antibody titers. Red circles: TBE patients without prior TBE vaccination (unvacc, *n* = 30; see Figure 1A,B). Blue triangles: TBE patients with a vaccination breakthrough infection (VBT, *n* = 11). (C) TBE neutralizing antibody titers. Red circles: TBE patients without prior vaccination (unvacc, *n* = 19; see Figure 1C). Blue triangles: TBE patients with a VBT infection (*n* = 11). (D) RB IgG antibody titers of the VBT serum pool without (mock depl.) and after depletion of broadly cross‐reactive antibodies with RB sE. (E) TBEV‐neutralizing antibody titers of the VBT serum pool without (mock depl.) and after depletion of broadly cross‐reactive antibodies with RB sE. (F) RB IgG antibody titers of three individual sera without (mock depl.) and after depletion of broadly cross‐reactive antibodies with RB sE. (G) TBEV‐neutralizing antibody titers of sera from TBE VBT patients without (mock depl.) and after depletion of broadly cross‐reactive antibodies with RB sE. The pools were tested in three independent experiments. Significances were determined with *t* tests (titers) or the Mann–Whitney test (ratios), and are indicated by asterisks (****p < 0.0001, **p = 0.01–0.001). Bars show the GMT (A, C) or median (B). Error bars show the standard error of the mean. Dotted line: cutoff of the assays. depl., depleted sera; ELISA, enzyme‐linked immunosorbent assays; GMT, geometric mean titer; IgG, immunoglobulin G; mock depl., mock‐depleted sera; ns, not significant; NT, neutralization test; sE, soluble E; TBE, tick‐borne encephalitis; TBEV, tick‐borne encephalitis virus; unvacc, unvaccinated.

TBE VBTs occur in rare instances^22^, ^26^ and most of these patients develop a strong anamnestic immune response.^23^, ^24^, ^25^ Since cross‐reactive antibodies have been implicated in immune‐enhancement phenomena such as ADE of DENV infections,^11^, ^20^ and a similar phenomenon was also speculated to contribute to cases of VBTs,^26^ we analyzed the patterns of type‐specific and cross‐reactive antibodies of TBE VBT patients (Table 3, Figure 5, Table S4). VBT patients developed significantly higher antibody titers than unvaccinated TBE patients, consistent with an anamnestic antibody response (Figure 5A). However, there was no significant difference with respect to the ratios of homologous and cross‐reactive antibody titers in the two groups (Figure 5B), indicating that antibody responses in VBTs had not shifted toward increased production of cross‐reactive antibodies. Consistent with the ELISA IgG titers, the TBE NT titers were significantly higher in the VBT group than in the unvaccinated group (Figure 5C). Similar to what has been observed in unvaccinated and YF‐prevaccinated TBE patients, the depletion of broadly flavivirus cross‐reactive antibodies from a pool of the 11 VBT sera as well as from single serum samples (Figure 5D,F) had no effect on TBEV neutralization (Figure 5E,G).

**Table 3:** Characteristics of TBE patients with prior vaccination.

TBE patients	No. of cases	Age (years)		Sex (f/m)
		Median	Range	
TBE vaccination breakthrough infection^a^	11	61	19‐73	2/9

Abbreviations: f, female; m, male; TBE, tick‐borne encephalitis.

^a^ Two patients were within the recommended vaccination schedule.

## 4 DISCUSSION

Co‐circulating flaviviruses and the use of different flavivirus vaccines increase the likelihood of multiple and sequential exposures to flavivirus antigens, which may influence the course and pattern of immune responses. In this study, we compared the patterns of antibody reactivities after TBEV infections of unvaccinated and YF‐prevaccinated patients as well as DEN patients with a prior TBE and/or YF vaccination. We show that a TBEV infection in unvaccinated individuals elicits only low levels of broadly cross‐reactive antibodies, as measured by ELISA with the sE protein of the distantly related RBV (Figure 2A,B). However, cross‐reactive epitopes are not completely silent in TBEV, because we found a significantly stronger induction of broadly cross‐reactive antibodies in YF‐prevaccinated TBE patients. As in a TBEV infection, YF vaccination of flavivirus‐naïve individuals elicits only low levels of cross‐reactive antibodies,^35^, ^43^ but the sequential encounter of conserved sites through antigens of both flaviviruses apparently results in strong stimulation of pre‐existing B memory cells, resulting in a boost of broadly cross‐reactive antibodies. Of note, no significant difference between RB and TBE sE IgG titers was found in the YF‐prevaccinated TBE cohort (Figure 2A). Since the TBE sE reacts with both type‐specific and cross‐reactive antibodies^12^, ^30^, ^42^ and the RB sE only with broadly cross‐reactive antibodies, these data indicate that type‐specific antibodies represent only a small fraction of the increased polyclonal antibody response in this cohort. Taken together, the data show that a pre‐existing YF immunity in TBE patients resulted in significantly higher antibody responses that are skewed toward cross‐reactive epitopes. However, no difference was observed in TBEV neutralization assays between unvaccinated and YF‐preimmune cohorts (Figure 2C), indicating that the pre‐existing YF immunity did not affect the induction of these functional antibodies and that neutralization of TBEV is primarily mediated by type‐specific antibodies.

The shift in the antibody repertoire toward cross‐reactive epitopes in patients with a history of heterologous flavivirus exposure also has practical implications for the use of ELISAs to analyze antibody responses after vaccination. In flavivirus‐experienced individuals, the functional antibody response can be grossly overestimated in ELISAs, because, at least for TBEV, cross‐reactive antibodies are not involved in virus neutralization. Indeed, as demonstrated in our study, no increase in neutralizing activity was observed in the YF‐preimmune TBE cohort (Figure 2C), consistent with the interpretation that primarily newly induced type‐specific antibodies neutralize TBEV. This is also in agreement with other reports in which TBEV strains are neither neutralized by heterologous DEN sera^44^ nor by broadly cross‐reactive monoclonal antibodies.^42^, ^45^ If individuals have different histories of pre‐existing immunity (which may be unknown to them), the proportions of neutralizing and nonneutralizing antibodies may differ substantially.

TBE‐ and/or YF‐prevaccinated DEN patients had significantly higher levels of cross‐reactive antibodies than YF‐prevaccinated TBE patients (∼26‐fold) (Figure 2). Additionally, we performed a comparison with published data from TBE‐ and/or YF‐prevaccinated Zika patients.^12^ Their serum samples were analyzed with the same IgG ELISA using RB sE as an antigen and the broadly cross‐reactive titers were also significantly lower (∼12‐fold) than those of the DEN patients (Figure S4),^12^ suggesting that DENV infections with prior heterologous immunity might be especially prone to elicit cross‐reactive antibodies. We found striking differences in the ability of cross‐reactive antibodies to neutralize TBE and DEN viruses, with TBEV being resistant to broad cross‐neutralization, and DENV being highly susceptible (Figures 3 and 4). These observations can be linked to differences in the structural properties of the two viruses. As the FL is the only region in E that is conserved across the viruses tested, it is likely that cross‐reactive antibodies react with epitopes comprising the FL, and the differences observed result from different FL exposures in TBE and DEN viruses (Figure 1).^8^ Although the FL is buried on the surface of mature virions, it can be more accessible in partially mature virions or become transiently exposed during virus breathing.^8^, ^9^, ^10^ Compared to TBE and Zika viruses, these phenomena appear to be more pronounced with DENV.^12^, ^46^ A stronger FL exposure in DENV can lead to (i) a greater dominance of the FL as an epitope and consequently stronger antibody responses than after TBE or Zika virus infections, and (ii) virus neutralization by FL‐specific cross‐reactive antibodies, because of the transient and/or partial exposure of the FL at the surface of infectious virus particles. Consistent with an increased exposure of the FL in DENV, substantial levels of FL‐specific antibodies were already detected after primary DENV infections in various studies.^17^, ^47^, ^48^, ^49^ It is important to emphasize, however, that the degree of FL exposure can vary between different DENV serotypes, strains, and/or preparations, leading to different potencies in virus neutralization,^50^, ^51^, ^52^, ^53^, ^54^, ^55^, ^56^, ^57^ with laboratory‐adapted strains being more susceptible than natural isolates.^53^, ^55^ These aspects should be taken into account when interpreting NT results, as meaningful comparisons of neutralization data are only possible under strictly standardized test conditions.

In the case of TBE VBTs, it has been speculated that immune‐enhancement phenomena might be involved in disease development, similar to DEN,^58^ but so far, there is no evidence for such a scenario in TBE VBTs.^26^ Since FL‐specific cross‐reactive antibodies have been shown to facilitate ADE of DENV infections of Fcγ receptor‐bearing cells and therefore appear to play an important role in the development of severe DEN,^11^, ^20^ we analyzed a possible shift toward cross‐reactive antibodies in TBE VBTs. Although we observed an overall stronger antibody response in VBTs, consistent with a booster reaction as described previously,^23^, ^24^, ^25^ we did not see a higher proportion of cross‐reactive antibodies relative to type‐specific neutralizing antibodies in VBTs. Therefore, our data do not support a similar role for cross‐reactive antibodies in severe TBE VBT disease as in DEN, which is most likely related to the structural differences between the two viruses. Consistent with our previous interpretation, it appears that the boosted neutralizing antibody response was too slow to prevent disease in these VBT cases.^23^ As type‐specific antibodies are responsible for efficient TBEV neutralization, our data also underline the high functional quality of antibodies induced in the course of VBTs, which is in agreement with previous reports.^23^, ^24^, ^25^

As our samples were not derived from a prospective study but were randomly received for diagnostic purposes, the groups analyzed were relatively heterogeneous with respect to age and comprised mostly adults. We therefore also looked specifically at the antibody responses of the few individuals with an age <18 years (Figures S5 and S6) and found that their titers were within the range of all values obtained. Also early blood withdrawal (<7 days after symptom onset) had no influence on titer development (Figure S7). A limitation of our study is that the time interval between prior TBE and/or YF vaccination and infection has not been reported to the diagnostic laboratory, which might affect the magnitude of IgG titers. Therefore, further investigations with a larger sample size and variable time span between different flavivirus exposures are needed to analyze this parameter.

In conclusion, we show that pre‐existing vaccine‐induced flavivirus immunity results in booster reactions that can substantially change antibody patterns formed in the course of flavivirus infections. These effects can have important practical consequences, especially when analyzing antibody responses by in vitro immunoassays in vaccine studies, because of a major impact on the correlation between ELISA and NT titers.

Our study highlights significant differences in the structural properties of different flaviviruses that affect the induction of broadly cross‐reactive antibodies and their functional activities in virus neutralization. We have analyzed selected cohorts of TBE and DEN patients, but the picture may become even more complex in other situations of different combinations of sequential flavivirus infections and/or vaccinations. Further studies of individuals with multiple flavivirus exposures may provide important additional insights into the complex antigenic structure of flaviviruses and their biological implications.

## AUTHOR CONTRIBUTIONS

*Conceptualization and design of the study*: Karin Stiasny. *Methodology and investigation*: Lena Roßbacher, Stefan Malafa, Melissa Thaler, and Stephan W. Aberle. *Data analysis*: Lena Roßbacher and Karin Stiasny. *Resources*: Kristina Huber, Stephan W. Aberle, and Judith H. Aberle. *Funding acquisition and supervision*: Karin Stiasny. *Writing of the manuscript*: Karin Stiasny, Franz X. Heinz, and Lena Roßbacher. All authors contributed to the article and approved the submitted version.

## CONFLICTS OF INTEREST STATEMENT

The Center for Virology received an Investigator‐Initiated Research grant from Pfizer (Project WI235042 TBE in Austria—Extended assessment of clinical course including sequelae and vaccination status of virologically verified TBE cases based on active surveillance), with Judith H. Aberle as principal investigator. Karin Stiasny and Franz X. Heinz are inventors on a patent of the Medical University of Vienna on flavivirus serodiagnosis. The remaining authors declare no conflict of interest.

## ETHICS STATEMENT

The studies were approved by the Ethics Committee of the Medical University of Vienna (EK number 1184/2020). Only leftover anonymized samples from routine laboratory diagnoses were analyzed in this retrospective study. No sample was specifically collected for this study. Patient consent was not necessary because of the use of leftover diagnostic samples in anonymized form.

## Supporting information

Supporting information.

## Data Availability

The data generated or analyzed during this study are included in this article and its supplementary information.
