# Supplementary material for: Effect of previous heterologous flavivirus vaccinations on human antibody responses in tick‐borne encephalitis and dengue virus infections
Source: J Med Virol. 2023 Nov 27;95(11):e29245. doi: 10.1002/jmv.29245 (PMC10952712; doi:10.1002/jmv.29245)
Supplement: Supplementary file 1 — Supporting information. [file JMV-95-0-s001.pdf]

Supporting information

Effect of previous heterologous flavivirus vaccinations on human antibody responses in tick-borne encephalitis and dengue virus infections

Lena Roßbacher 1, Stefan Malafa 1, Kristina Huber 2, Melissa Thaler 1,3, Stephan W. Aberle 1, Judith H. Aberle 1, Franz X. Heinz 1, Karin Stiasny 1*

1 Center for Virology, Medical University of Vienna, Vienna, Austria

2 Division of Infectious Diseases and Tropical Medicine, University Hospital, LMU Munich, Munich, Germany

3 Present address: Department of Medical Microbiology, Leiden University Medical Center, Leiden, Netherlands

Supporting Table 1

Time interval between samples obtained from TBE patients and symptom onset

TBE patients	No. of cases[a]	Days post symptom onset	
		Median	Range
Unvaccinated	23	14	3-31
Prior YF vaccination	8	14	5-26

[a] No. of cases for which symptom onset was reported to the diagnostic laboratory

Supporting Table 2

Time interval between samples obtained from DEN patients and symptom onset

DEN patients	No. of cases[a]	Days post symptom onset		Interval between symptom onset and PCR (days)	
		Median	Range	Median	Range
Prior TBE vaccination	8	11	8-30	3	2-6
Prior YF vaccination	1	7	-	0	0
Prior TBE+YF vaccination	7	11	6-19	5	1-6

[a] No. of cases for which symptom onset was reported to the diagnostic laboratory

Supporting Table 3

Serum samples from TBE and DEN patients used for pools

Pools	n[a]
Unvaccinated TBE patients	19
YF-pre-vaccinated TBE patients	10
TBE-pre-vaccinated DEN[b] patients	12
TBE/YF-pre-vaccinated DEN[b] patients	8

[a] Number of patient samples included in pools (one sample per patient). [b] Only DENV-serotype-2-infected patients were included.

Supporting Table 4

Time interval between samples obtained from TBE patients with prior TBE vaccination and symptom onset

TBE patients	No. of cases[a]	Days post symptom onset	
		Median	Range
TBE vaccination breakthrough infection (VBT)	8	14	5-26

[a] No. of cases for which symptom onset was reported to the diagnostic laboratory

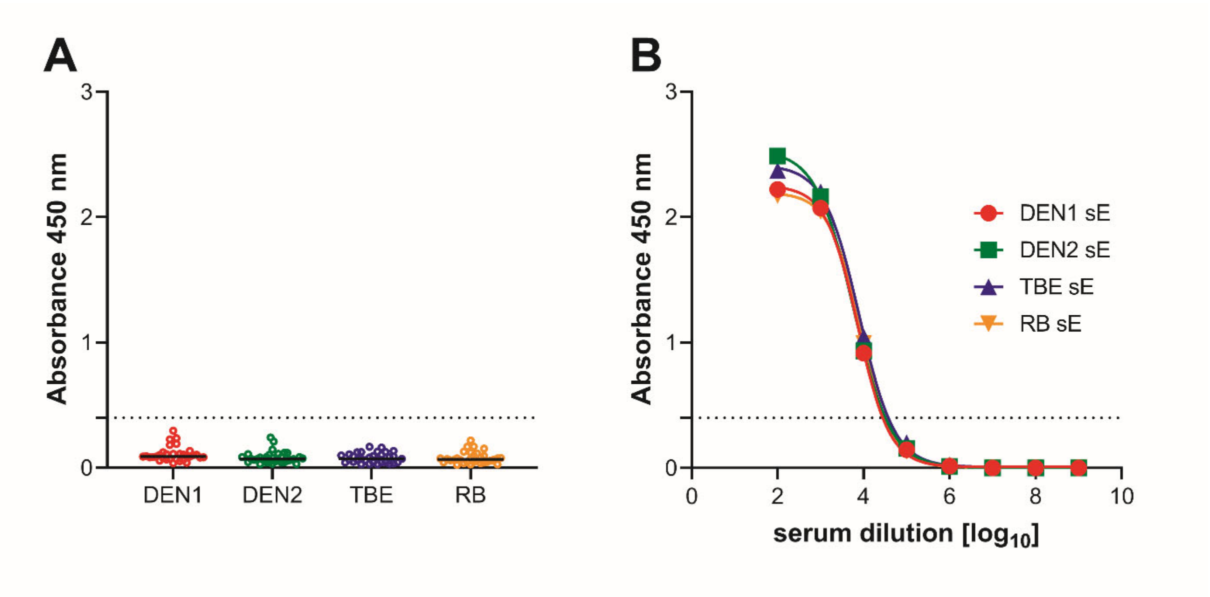

Supporting Figure 1: Flavivirus IgG ELISA controls. (A) ELISA absorbance values of 32 flavivirus-negative control sera (dilution 1:100) with DEN1, DEN2, RB and TBE virus sE proteins. (B) Titration curves of the flavivirus cross-reactive positive control serum with different sE antigens used in this study (as described in Material and Methods).

The cut-off used for titer calculations is shown as dotted line in both panels. DEN, dengue; RB, Rio Bravo; sE, soluble E; TBE, tick-borne encephalitis.

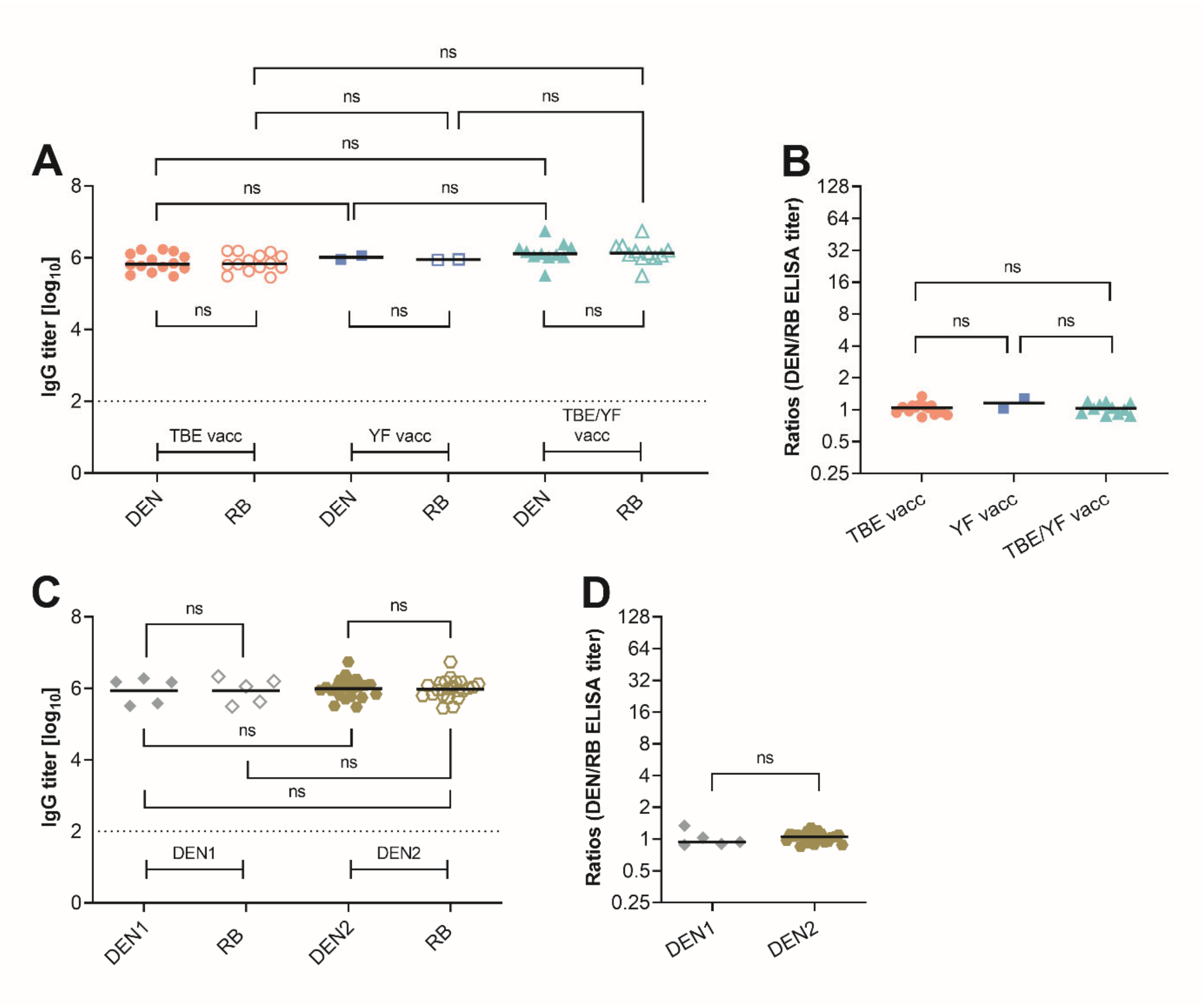

Supporting Figure 2: IgG antibody titers directed to the homologous DEN sE or heterologous RB sE protein, stratified by flavivirus pre-exposure history (A,B) and DEN serotype (C,D). (A, C) IgG titers, determined with the homologous sE, are shown as filled symbols; broadly cross-reactive IgG titers, determined with RB sE, as empty symbols. (B, D) Ratios of homologous to broadly cross-reactive antibodies.

Pink circles: DEN patients with prior TBE vaccination (TBE vacc n = 14); light blue squares: DEN patients with a prior YF vaccination (YF vacc, n = 2); mint triangles: DEN patients with prior TBE and YF vaccination (TBE/YF vacc, n = 11). Grey diamonds: DEN 1 patients with a prior TBE and/or YF vaccination (DEN1, n = 5); gold hexagons: DEN 2 patients with a prior TBE and/or YF vaccination (DEN2, n = 22).

Bars show the GMT (A,C) or median (B,D). Significant differences between the titers of the three groups were determined by one-way ANOVA followed by Bonferroni's or Tukey's multiple comparison test, between ratios with the Kruskal Wallis test followed by Dunn's multiple comparison test or the Mann-Whitney test. Significant differences within the groups were determined by t-tests. Dotted line: cut-off of the assays. DEN, dengue; ns, not significant; RB, Rio Bravo; TBE, tick-borne encephalitis; vacc, vaccinated; YF, yellow fever.

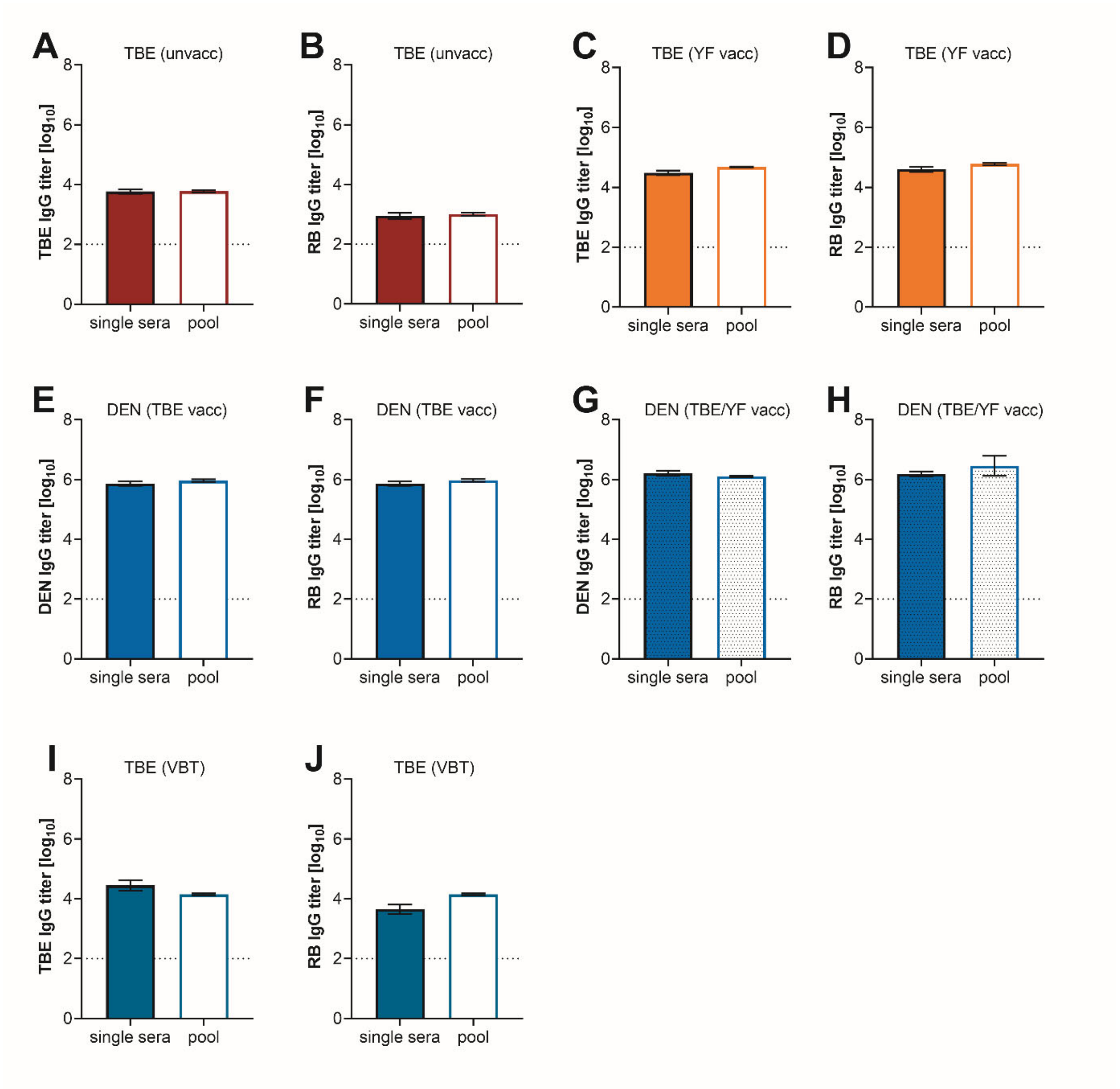

Supporting Figure 3: Comparison of GMTs from single sera and the respective serum pools from TBEV- and DENV-serotype-2-infected patients. The GMT of single serum samples is shown as filled columns, the GMT of the pools as empty columns.

(A) Homologous and (B) broadly cross-reactive IgG antibody titers of TBE patients without a prior TBE or YF vaccination [TBE (unvacc)], measured with TBE sE and RB sE, respectively. (C) Homologous and (D) broadly cross-reactive IgG antibody titers of TBE patients with a prior YF vaccination [TBE (YF vacc)], measured with TBE sE and RB sE, respectively. (E) Homologous and (F) broadly cross-reactive IgG antibody titers of DEN patients with a prior TBE vaccination [DEN (TBE vacc)], measured with DEN sE and RB sE, respectively. (G) Homologous and (H) broadly cross-reactive IgG antibody titers of DEN patients with a prior TBE and YF vaccination [DEN (TBE/YF vacc)], measured with DEN sE and RB sE, respectively. (I) Homologous and (J) broadly cross-reactive IgG antibody titers of vaccination-breakthrough (VBT) TBE patients [TBE (VBT)], measured with TBE sE and RB sE, respectively. The pools were tested in three independent experiments. No significant differences were found with t-tests between the GMT of single serum samples and the respective pools. Error bars show the standard error of the mean. Dotted line: cut-off of the assays. DEN, dengue; RB, Rio Bravo; TBE, tick-borne encephalitis; vacc, vaccinated; YF, yellow fever.

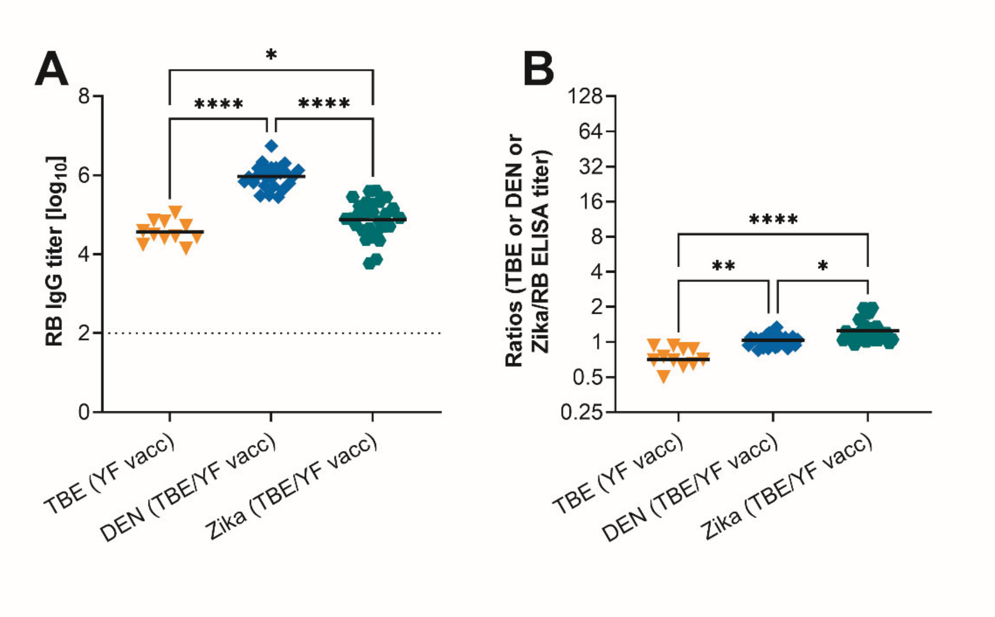

Supporting Figure 4: Comparison of broadly cross-reactive IgG antibodies in TBE and DEN post-infection sera with those in Zika post-infection sera.

The titers of the Zika post-infection sera are from Malafa et al, 2020 (17 with a prior TBE vaccination, 4 with a prior YF vaccination, 8 with prior TBE and YF vaccination). The samples were obtained at a median of 14 days after disease onset (range 7-30 days).

(A) Broadly cross-reactive IgG titers were determined with RB sE. (B) Ratios of homologous to broadly cross-reactive antibodies. Orange triangles: Flavivirus TBE patients with a prior YF vaccination [TBE (YF vacc), n = 11]; blue diamonds: DEN patients with prior TBE and/or YF vaccination [DEN (TBE/YF vacc), n=27]; green hexagons: Zika patients with prior TBE and/or YF vaccination [Zika (TBE/YF vacc), n=29]. Bars show the GMT (A) or median (B).

Significant differences between the titers of the three groups were determined by one-way ANOVA followed by Bonferroni's multiple comparison test, between ratios with the Kruskal Wallis test followed by Dunn's multiple comparison test. Significant differences within the groups were determined by t-tests. Significances are indicated by asterisks (**** $p < 0.0001$, ** $p = 0.001$ to 0.01, * $p = 0.01$ to 0.05). Dotted line: cut-off of the assays. DEN, dengue; ns, not significant; RB, Rio Bravo; sE, soluble E; TBE, tick-borne encephalitis; vacc, vaccinated; YF, yellow fever.

Malafa S, Medits I, Aberle JH, et al. Impact of flavivirus vaccine-induced immunity on primary Zika virus antibody response in humans. PLoS Negl Trop Dis. 2020;14(2):e0008034.

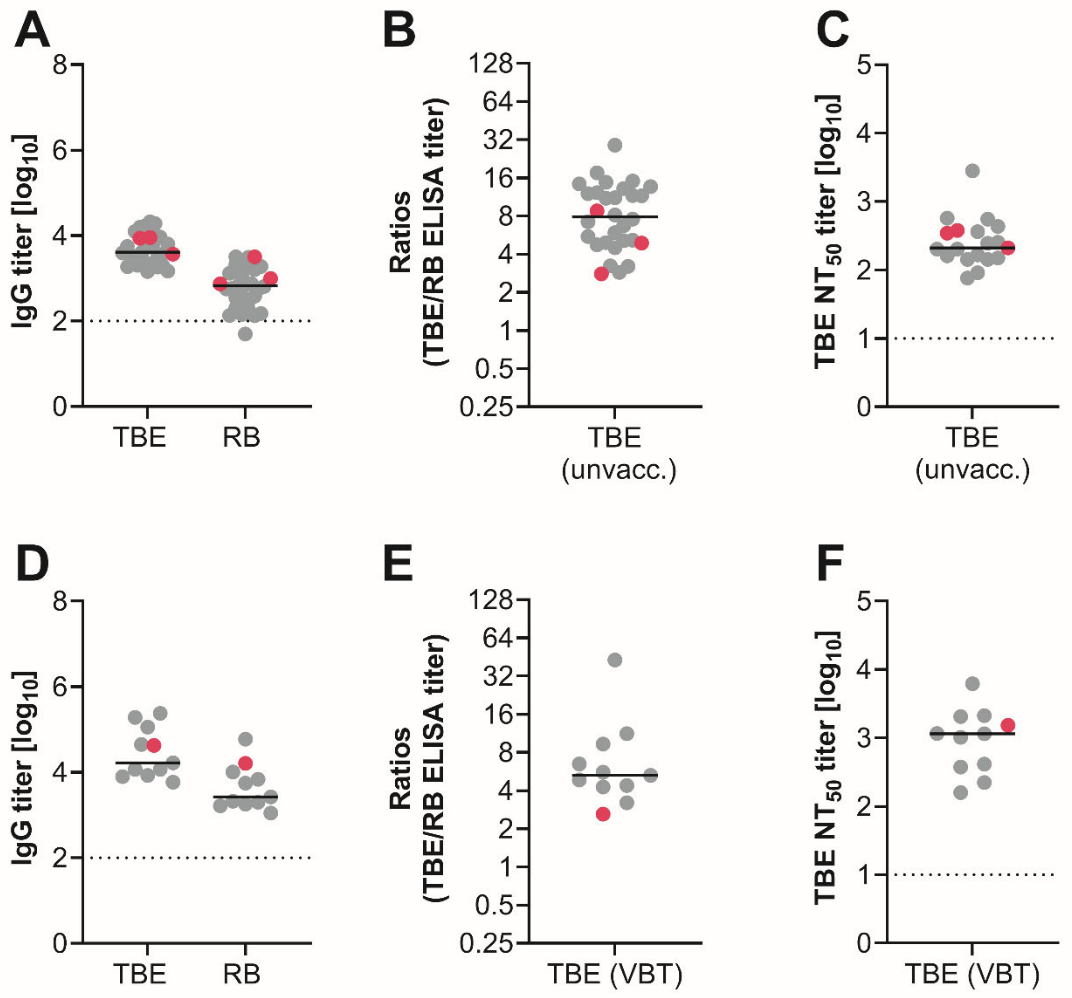

Supporting Figure 5: Antibody responses of TBE patients without a prior TBE or YF vaccination (A-C), and TBE-vaccination breakthrough patients (VBT) (D-F). Values from individuals who were under 18 years old at the time of their TBE infection are shown in pink. (A-C) n=3. (D-F) n=1.
(A,D) IgG titers, determined with homologous sE (TBE), and broadly cross-reactive IgG titers, determined with Rio Bravo sE. (B,E) Ratios of homologous to broadly cross-reactive antibody titers. (C,F) TBE NT titers. Dotted line: cut-off of the assays. NT, neutralization test; RB, Rio Bravo; sE, soluble E; TBE, tick-borne encephalitis; unvacc, unvaccinated; YF, yellow fever.

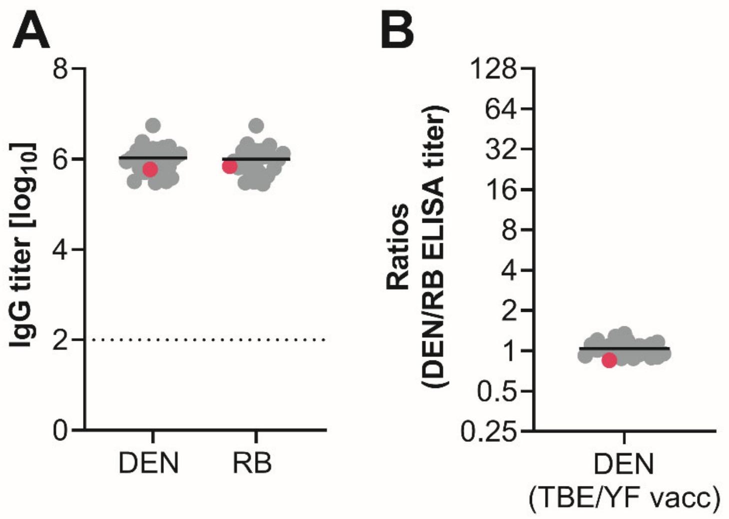

Supporting Figure 6: Antibody responses of TBE- and/or YF-pre-vaccinated DEN patients. Values from individuals who were under 18 years old at the time of their DENV infection are shown in pink (n=1). (A) IgG titers, determined with homologous sE (DEN), and broadly cross-reactive IgG titers, determined with Rio Bravo sE. (B) Ratios of homologous to broadly cross-reactive antibody titers. Dotted line: cut-off of the assay. DEN, dengue; RB, Rio Bravo; sE, soluble E; TBE, tick-borne encephalitis; vacc, vaccinated; YF, yellow fever.

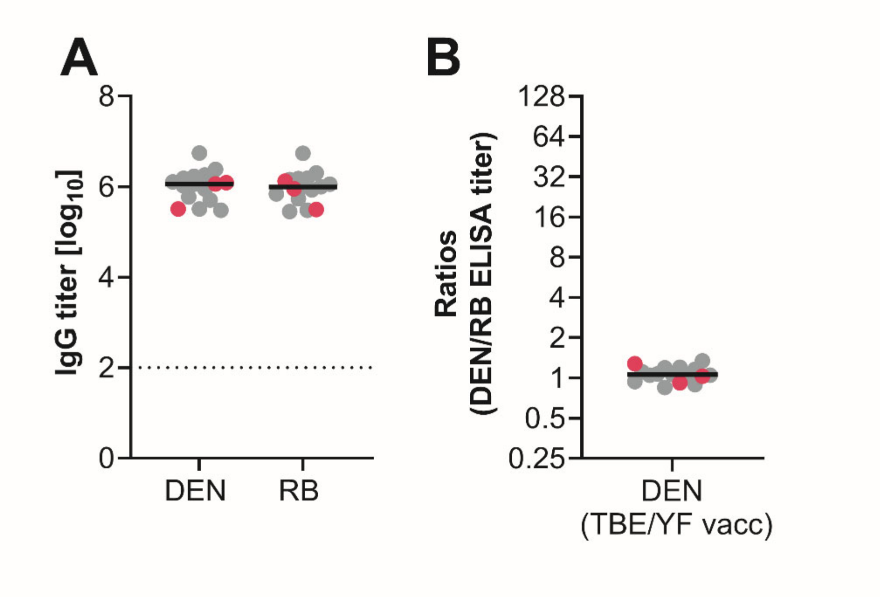

Supporting Figure 7: Antibody responses of the TBE- and/or YF-pre-vaccinated DEN individuals with known interval between symptom onset and blood sampling (n=16). Values from individuals whose disease onset was less than 7 days prior to their blood withdrawal are shown in pink (n=3).
(A) IgG titers, determined with homologous sE (DEN), and broadly cross-reactive IgG titers, determined with Rio Bravo sE. (B) Ratios of homologous to broadly cross-reactive antibody titers. Dotted line: cut-off of the assay. DEN, dengue; RB, Rio Bravo; sE, soluble E; TBE, tick-borne encephalitis; vacc, vaccinated; YF, yellow fever.
